# Curcumin ameliorates hepatic insulin resistance by activating PINK1/Parkin-mediated mitophagy

**DOI:** 10.1038/s41598-026-47924-6

**Published:** 2026-04-08

**Authors:** Zhanhong Guo, Huan Zhang, Yinghong Kong

**Affiliations:** 1 Department of Endocrinology, Affiliated Changshu Hospital of Nantong University, Changshu, 215500 Jiangsu China; 2 Department of General Practice, Affiliated Changshu Hospital of Nantong University, Changshu, 215500 Jiangsu China

**Keywords:** Curcumin, Insulin resistance, Mitophagy, PINK1/Parkin, HepG2 cells, Biochemistry, Cell biology, Diseases, Molecular biology

## Abstract

Insulin resistance (IR) is the key pathogenic factor of type 2 diabetes (T2DM) and its associated metabolic diseases. Mitochondrial dysfunction and inadequate mitophagy are central to this pathology. Curcumin, the main bioactive compound of *Curcuma longa*, has been reported to enhance insulin sensitivity, yet the mechanism in hepatocytes remains debated. Here we investigated whether curcumin alleviates hepatocyte IR by targeting PTEN-induced putative kinase 1 (PINK1)/ Parkin-dependent mitophagy. We induced IR in HepG2 cells with palmitic acid (PA) and then treated with curcumin. To verify the mechanism, we used PINK1 siRNA (siPINK1) and the mitophagy blocker Mdivi-1. Glucose uptake, glycogen content, lipid accumulation, phosphatidylinositol 3-kinase (PI3K)/ protein kinase B (Akt)/ glucose transporter 4 (GLUT4) signaling, mitochondrial function, and mitophagy markers were quantified by flow cytometry, glycogen assay kit, Oil Red O staining, confocal imaging, and Western blot. Curcumin mitigated PA-induced IR, increased PI3K/Akt phosphorylation and GLUT4 expression, promoted GLUT4 translocation to the plasma membrane, restored mitochondrial membrane potential and ATP content, and enhanced PINK1/Parkin-mediated mitophagy. These effects were partially blunted by siPINK1 or Mdivi-1. In HepG2 cells, curcumin improves insulin resistance by activating PINK1/Parkin-dependent mitophagy and rescuing mitochondrial function.

**Supplementary Information:**

The online version contains supplementary material available at 10.1038/s41598-026-47924-6.

## Introduction

T2DM is characterized by insulin resistance and insufficient insulin secretion, and has become a significant global public health challenge. The Global Burden of Disease study estimated that over 500 million people were living with diabetes in 2021, with this number expected to rise through 2050^1^. Natural compounds act on multiple pathways with minimal toxicity and high tolerability, distinguishing them from traditional antidiabetic drugs. Growing research focuses on their effects in anti-inflammatory, antioxidant, and metabolic reprogramming pathways^2^. Plant-derived bioactives have been observed to enhance insulin sensitivity and stabilize glucose control^3,4^.

The liver plays a major role in glucose homeostasis through dynamic regulation of glycolysis, gluconeogenesis, glycogen synthesis and fatty acid oxidation^5^. Excessive oxidative activity of mitochondria caused by high glucose levels can lead to overproduction of reactive oxygen species (ROS), resulting in consequent mitochondrial damage and lipid accumulation, including diacylglyceroles and ceramides, which directly disrupt tyrosine phosphorylation of the insulin receptor substrate (IRS) proteins. Such interference inhibits the PI3K/Akt signaling and leads to hepatic insulin resistance^6^. Additionally, high glucose inhibits fatty acid β-oxidation and promotes de novo lipogenesis, increasing lipotoxic consequences and overloading the mitochondria^7^.

Several compounds, such as resveratrol, berberine and metformin, have been shown in preclinical models to reverse hepatic insulin resistance^8–10^. Curcumin, the main bioactive compound of *Curcuma longa*, exhibits multiple pharmacological activities, including antioxidant, immunomodulatory and antifibrotic properties. It shows potential benefits for various diseases, such as joint diseases, intestinal inflammation, cardiovascular and liver diseases^11,12^. Recent evidence indicates that curcumin improves insulin resistance in T2DM patients^13^. However, the underlying molecular mechanisms remain unclear. Curcumin has significantly low oral bioavailability with a rapid systemic clearance, which limits its therapeutic use in clinical practice^14^. Nevertheless, its hydrophobicity may actually enable regulation of membrane-related signaling pathways, potentially enriching it in lipid bilayers and mitochondrial membranes. Recent study revealed that curcumin stabilizes PINK1 and promotes the recruitment of Parkin to mitochondria^15^.Therefore, its physicochemical limitations may become a pharmacological advantage by targeting mitochondrial quality control pathways.

Mitophagy plays an essential role as a quality control process by selectively removing damaged mitochondria^16^. This maintains cellular energy production and redox balance^16^. Studies have found that impaired mitochondrial quality control aggravates insulin resistance, forming a vicious cycle^17^. Therefore, restoring mitophagy provides a potential therapeutic strategy. Natural products are increasingly linked to insulin-sensitizing effects through activation of PINK1/Parkin-dependent mitophagy^18^. Whether curcumin engages this pathway to counter insulin resistance remains uncertain, and the connections among curcumin, mitophagy, and insulin signaling require clarification.

Although interest in natural mitophagy agonist is growing, no prior study has directly linked the insulin-sensitizing effects of curcumin to PINK1-mediated mitophagy in the hepatocytes. Researchers have reported the ability of curcumin to improve insulin resistance but attributed this effect to c-Jun N-terminal kinase (JNK) inhibition^19,20^ or nuclear factor erythroid 2-related factor 2 (Nrf2) activation^20^. Here, we demonstrate that PINK1-dependent mitophagy is a core mechanism through which curcumin improves insulin resistance in hepatocytes.

Based on the above research, we propose that curcumin reverses insulin resistance by activating mitophagy. Our study shows for the first time that curcumin can improve insulin resistance by activating mitophagy mediated by the PINK1/Parkin signaling pathway, providing potential options for the treatment of insulin resistance.

## Methods

### Cell culture and treatment

HepG2 cells were obtained from the Cell Bank of the Chinese Academy of Sciences. Cells were maintained in DMEM (ThermoFisher, USA) supplemented with 10% FBS (ThermoFisher, USA) and 1% penicillin-streptomycin (Sangon Biotech, China) at 37 °C with 5% CO_2_. After IR induction, cells were treated with curcumin (10 µM, Sigma-Aldrich, USA) with or without Mdivi-1 (50 µM, MedChemExpress, USA) for 48 h, followed by a 30-minute exposure to insulin (100 nM).

### Construction of the insulin resistance model

Upon reaching 50% confluency, cells was switched to DMEM containing PA (0.25 mM, MedChemExpress, USA) and 25 mM glucose and maintained for 24 h to induce insulin resistance. Control wells received the same DMEM but with 5.5 mM glucose and no PA.

### Cell transfection

Cells were divided into two groups: IR + Cur+siPINK1 and IR + Cur+siNC. For each group, complexes of 100 pM siPINK1 or siNC with 4 µL Lipo8000 (Beyotime Biotechnology, China) were added and incubated for 6 h. The medium was then replaced with fresh medium, and culture was continued for an additional 48 h.

### CCK-8 assay

Cells were seeded into 96-well plates. When cell density reached approximately 80%, cells were treated as described above. Following PBS washes, 100 µL DMEM with 10% CCK-8 reagent (Beyotime Biotechnology, China) was added and incubated for 1 h. The optical density was recorded by multifunctional reader (PerkinElmer, USA) at 450 nm.

### Glycogen content assay

Cells were harvested and lysed on ice. Supernatants were obtained after centrifugation. Glycogen content was detected using a glycogen assay kit (Solarbio Life Sciences, China) according to the manufacturer’s protocol. Optical density was measured at 620 nm on multifunctional reader (PerkinElmer, USA). Glycogen content was calculated and expressed as µg/10^6^ cells.

### Glucose uptake assay

2-NBDG is a fluorescent analog of glucose commonly used to examine cellular glucose uptake. Cells were cultured in serum-free medium with 2-NBDG (100 µM, Beyotime Biotechnology, China) for 30 min. After removing the dye solution, cells were washed twice with cold PBS and analyzed by flow cytometry (DxFLEX, Beckman Coulter, USA). Excitation and emission were set at 488 nm and 530 nm respectively (FL1 channel).

### Oil red o staining

Cells were washed with PBS and fixed with 4% paraformaldehyde (PFA) for 20 min. Following fixation, cells were washed with 1 mL of 60% isopropanol for 5 min. Isopropanol was then removed, and 1 mL of Oil Red O working solution was added to each well, followed by incubation at 37 °C for 20 min. Excess dye was removed by washing cells with PBS until the washes ran clear. Cells were stained with hematoxylin for 2 min and washed with PBS to remove excess stain. Images were captured using an upright microscope (ECLIPSE Ni-U, Nikon, Japan).

### GLUT4 immunofluorescence

Cells were incubated with 4% paraformaldehyde at room temperature for 15 min and fixed with 0.1% Triton X-100 for 10 min. Cells was blocked using 5% bovine serum albumin and incubated with GLUT4 primary antibody (Sanying Proteintech, China) overnight at 4 °C. Cells were washed three times with PBS and incubated with Alexa Fluor 488-conjugated secondary antibody (Sanying Proteintech, China) for 1 h at room temperature in the dark. Images were captured using a laser confocal microscope (FV1000, Olympus, Japan).

### ROS assay

Cell pellets were washed with PBS and resuspended with 1 mL of H2DCFDA (10 µM, MedChemExpress, USA) and incubated in the dark at 37 ℃. The vehicle control group received an equal volume of PBS instead of H2DCFDA. After incubation, cells were washed with PBS to remove excess dye. Flow cytometry (DxFLEX, Beckman Coulter, USA) was used for detection. The FL1 channel was used to record the fluorescence signal. The excitation and emission were set at 488 nm and 530 nm respectively. A minimum of 10,000 events were acquired within the cell gate based on FSC-A/SSC-A properties.

### Mitochondrial membrane potential assay

Cells were stained with 1 mL JC-1 working solution (Solarbio Life Sciences, China) for 20 min at 37 ℃ in darkness. After PBS washes, mitochondrial membrane potential was detected via flow cytometry (DxFLEX, Beckman Coulter, USA). Green fluorescence (monomer form of JC-1) was detected in FL1 channel and red fluorescence (aggregate form of JC-1) in FL2 channel. A minimum of 10,000 events were acquired within the cell gate based on FSC-A/SSC-A properties.

### Western blot analysis

Cells were lysed in cold RIPA buffer (Beyotime Biotechnology, China) supplemented with 1 mM phenylmethylsulfonyl fluoride (PMSF, Beyotime Biotechnology, China) and protease and phosphatase inhibitor cocktail (NCM Biotech, China) for 30 min on ice. Lysates were centrifuged at 12,000 g for 15 min at 4 °C and supernatant protein concentrations were quantified using the BCA assay (Macklin Reagent, China). Equal amounts of protein (30 µg per lane) were separated by 10% SDS-PAGE and transferred to 0.45 μm PVDF membranes (Beyotime Biotechnology, China) at 100 V for 90 min. Membranes were blocked with 10% BSA for 1 h and incubated overnight using the following primary antibodies (Cell Signaling Technology, USA): anti-p-PI3K (Tyr458), anti-PI3K, anti-p-Akt (Ser473), anti-Akt, anti-GLUT4, anti-PINK1, anti-Parkin, anti-p62, and anti-Actin. After washing with TBST, membranes were incubated with HRP-conjugated secondary antibodies for 1 h. Signals were developed by ECL and detected with a multi-functional imaging system (Tanon Science & Technology, China).

### Mitochondria-lysosome colocalization

Cells were incubated with Mito-Tracker Red CMXRos (200 nM, Beyotime Biotechnology, China), Lyso-Tracker Green (75 nM, Beyotime Biotechnology, China), and DAPI (10 µg/ml, Beyotime Biotechnology, China) for 30 min at 37 °C to label mitochondria, lysosomes and nucleus respectively. Cells were washed three times with the PBS to eliminate the remaining dye. Images were captured using a laser confocal microscope (FV1000, Olympus, Japan) and merged with ImageJ software. Manders colocalization coefficients were calculated to evaluate colocalization.

### Cellular ATP measurement

ATP content was determined by ATP assay kit (Solarbio Life Sciences, Beijing, China). Cells were seeded and processed according to manufacturer’s protocol. Relative light unit (RLU) was measured using a chemiluminescence detector. The BCA method was used to determined protein concentration. ATP content was normalized to protein amount.

### Oxygen consumption rate (OCR) measurement

Mitochondrial respiration was assessed by an Agilent Seahorse XF24 Analyzer (Agilent Technologies, USA). Cells were seeded and cultured for 24 h. Following probe hydration and a 1 h equilibration in a CO_2_-free incubator, OCR was recorded under sequential additions of Oligomycin (1.5 µM, Nonin Biological Technology, China), FCCP (1.0 µM, Selleck Chemicals, USA), and Rotenone/Antimycin A (0.5 µM, Maokang Biotechnology, China). Basal OCR was measured before oligomycin addition. Oligomycin was injected at 15 min. FCCP, a mitochondria uncoupler, was injected at 35 min to determine maximal OCR. Rotenone/Antimycin A were added at 55 min to determine non-mitochondrial respiration.

### Statistical analysis

GraphPad Prism 9 was utilized for statistical analysis. One-way ANOVA and Tukey’s honestly significant difference (HSD) were applied to determine the statistical significance between different groups. All experiments were repeated at least three times independently. *P* < 0.05 was considered statistically significant.

## Results

### Curcumin improves insulin sensitivity in HepG2 cells

**Fig. 1:**
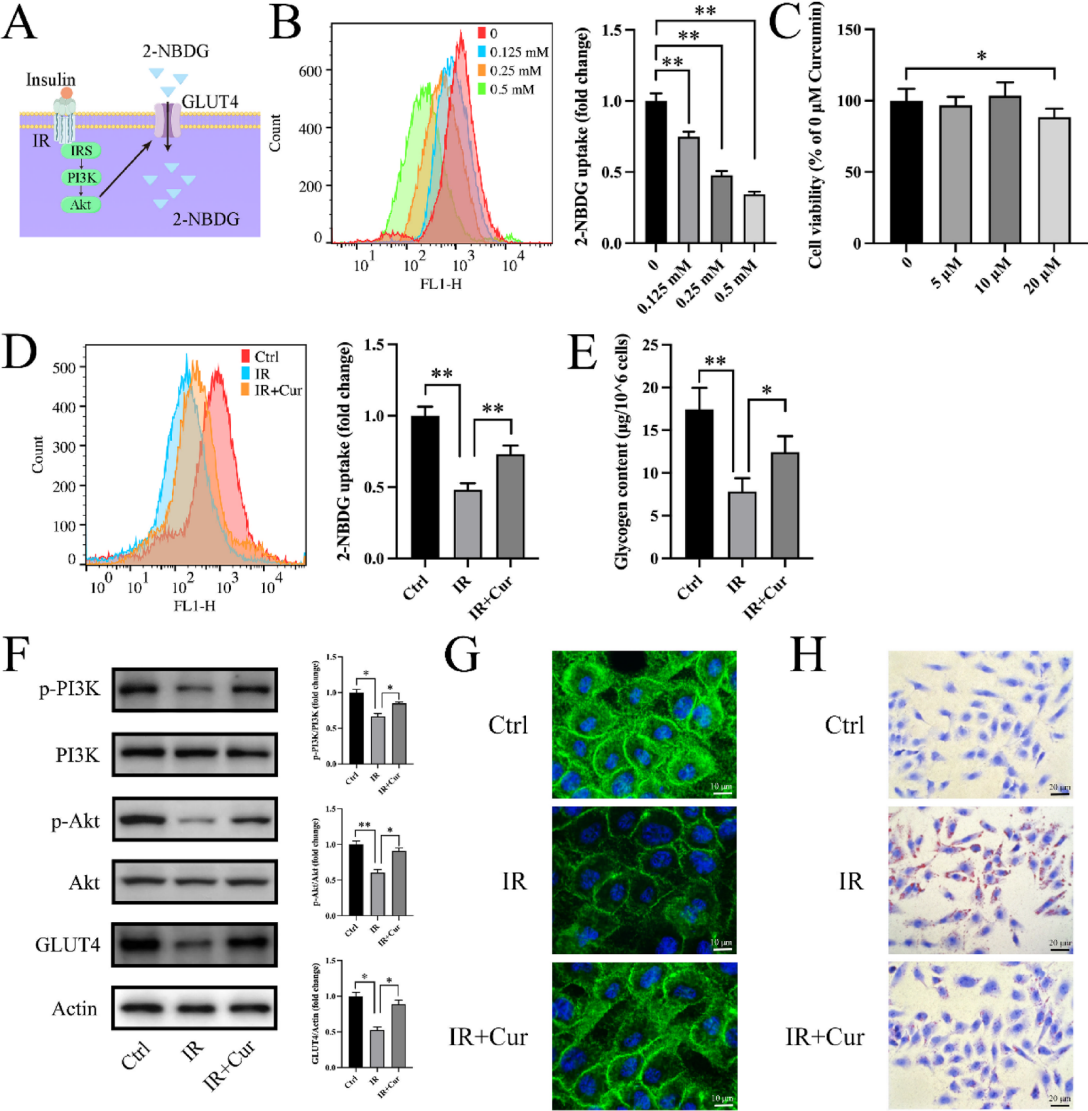
Effects of curcumin on insulin resistance. (**A**) Schematic diagram of insulin promoted 2-NBDG uptake. (**B**) Effects of different concentrations of PA on 2-NBDG uptake (*n* = 3). (**C**) Effects of different concentrations of curcumin on cell viability (*n* = 6). (**D**) Effects of curcumin on 2-NBDG uptake (*n* = 3). (**E**) Effects of curcumin on intracellular glycogen content (*n* = 3). (**F**) PI3K/Akt phosphorylation and GLUT4 expression (*n* = 3). (**G**) Immunofluorescence images show GLUT4 localization in both the cytoplasm and the plasma membrane. (H) Oil Red O staining shows lipid accumulation in HepG2 cells. (Tukey’s HSD, * *P* < 0.05, ** *P* < 0.01)

We assessed glucose metabolism in HepG2 cells. 2-NBDG is a fluorescent deoxyglucose analog used to directly detect glucose uptake in living cells (Fig. 1A). To establish the insulin resistance model, HepG2 cells were treated with 0.25 mM PA combined with high glucose. We found that 0.25 mM PA significantly reduced 2-NBDG uptake compared to control cells (Fig. 1B), consistent with previous studies^21^. Compared with the Ctrl group, cells after PA treatment showed obvious insulin resistance, as evidenced by significant decreases in 2-NBDG uptake (48.2 ± 4.49% of Ctrl group, Fig. 1D) and intracellular glycogen content (44.9 ± 9.03% of Ctrl group, Fig. 1E). A concentration of 10 µM curcumin was selected based on CCK-8 assays showing no cytotoxicity up to 10 µM, whereas 20 µM significantly reduced cell viability (Fig. 1C). Curcumin treatment significantly increased 2-NBDG uptake (152 ± 12.4% of IR group, Fig. 1D) and restored glycogen content (159 ± 24.1% of IR group, Fig. 1E). The phosphorylation of PI3K/Akt and expression level of GLUT4 were increased after curcumin treatment (Fig. 1F). Considering that PI3K signaling promotes GLUT4 translocation to the plasma membrane, we examined GLUT4 localization and expression by immunofluorescence. Curcumin promoted GLUT4 expression and cell membrane transposition (Fig. 1G). Additionally, Oil Red O staining results showed that curcumin significantly reduced intracellular lipid accumulation induced by PA (Fig. 1H).

### Curcumin attenuates PA-induced mitochondrial dysfunction in HepG2 cells

**Fig. 2:**
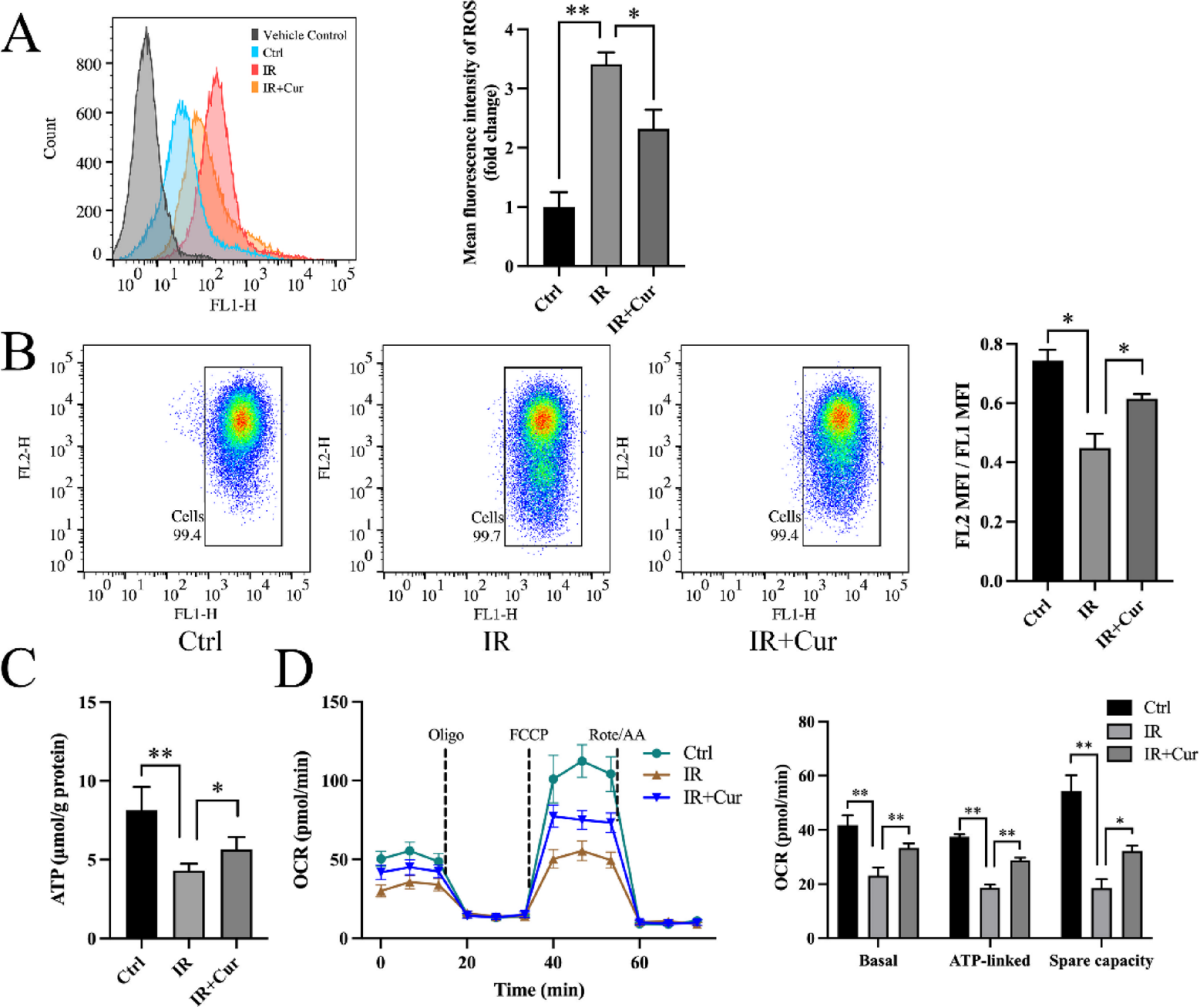
Effects of curcumin on mitochondrial homeostasis (*n* = 3). (**A**) ROS content in cells. (**B**) Mitochondrial membrane potential. (**C**) ATP content in cells. (**D**) Cell oxygen consumption rate. (Tukey’s HSD, * *P* < 0.05, ** *P* < 0.01)

To explore the impact of curcumin on mitochondrial function, we used flow cytometry to detect intracellular ROS production. Results demonstrated that PA treatment significantly increased ROS production (341 ± 19.7% of Ctrl group, Fig. 2A). Curcumin markedly reduced PA-induced ROS production (68.0 ± 9.35% of IR group, Fig. 2A). PA exposure markedly decreased mitochondrial membrane potential (ΔΨm), while curcumin reversed this change (Fig. 2B). Furthermore, PA reduced intracellular ATP levels (52.8 ± 5.43% of Ctrl group, Fig. 2C) and decreased OCR (Fig. 2D), and curcumin treatment partially reversed these effects (Fig. 2C, D). These results suggest curcumin exerts protective effects on mitochondrial function and energy metabolism.

### Curcumin activates PINK1-dependent mitophagy

**Fig. 3:**
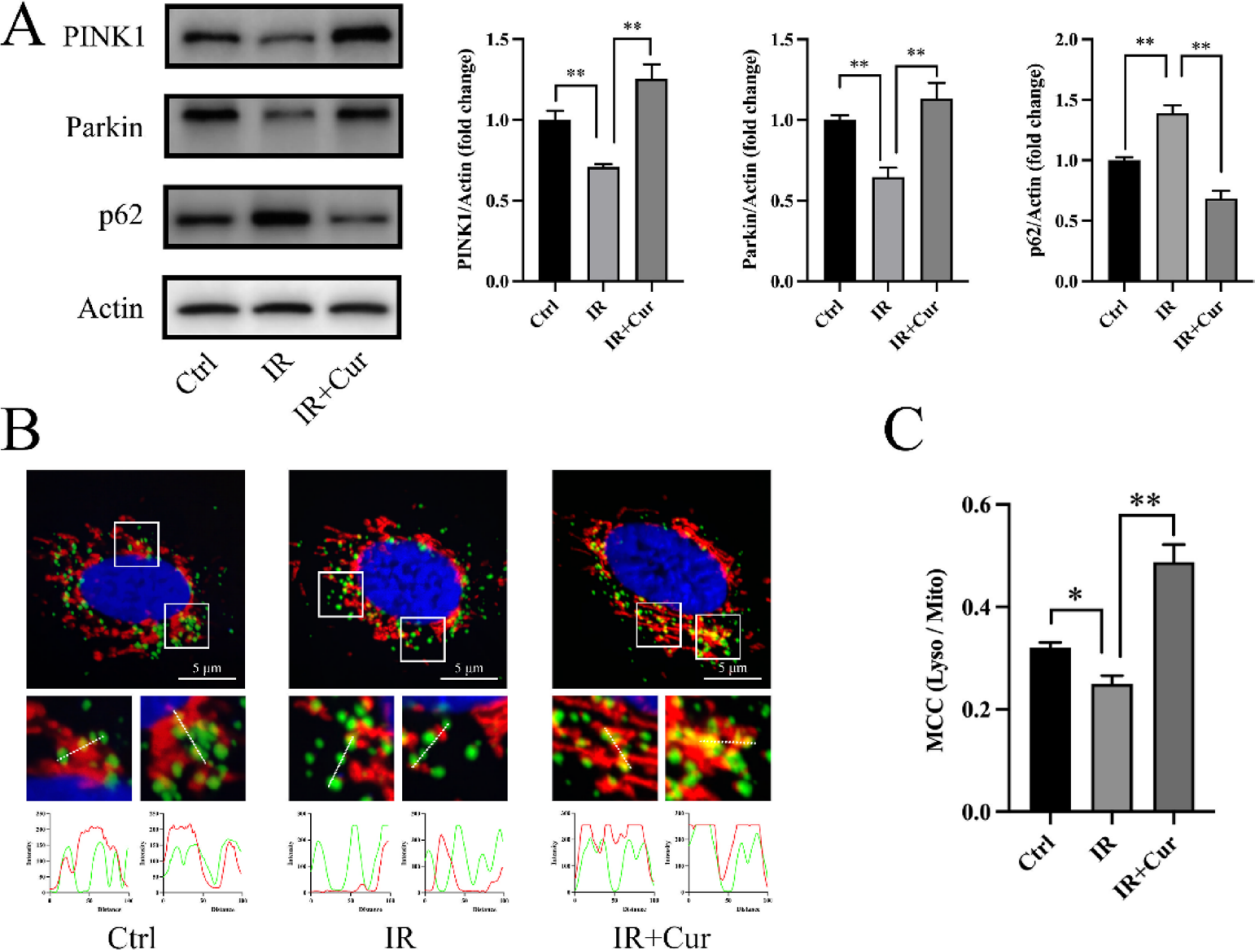
Effects of curcumin on mitophagy (*n* = 3). (**A**) Protein expression levels of PINK1/Parkin/p62. (**B**) Colocalization of mitochondria and lysosomes. (**C**) Manders’ colocalization coefficient of lysosomes and mitochondria. (Tukey’s HSD, * *P* < 0.05, ** *P* < 0.01)

We detected the expression of PINK1, Parkin and p62 via Western blot to assess PINK1-mediated mitophagy. The results revealed that PA significantly reduced PINK1 and Parkin protein levels, while increasing p62 expression (Fig. 3A), suggesting that PINK1-dependent mitophagy was inhibited. Curcumin partially restored these changes induced by PA (Fig. 3A). We visualized colocalization of mitochondria and lysosomes by laser confocal microscopy. PA reduced the overlap of mitochondrial and lysosomal fluorescence signals (Fig. 3B) and the Manders’ colocalization coefficient (77.9 ± 5.01% of Ctrl group, Fig. 3C), suggesting decreased mitophagy under insulin resistant conditions. Curcumin treatment significantly restored colocalization (195 ± 13.6% of IR group, Fig. 3B-C). In summary, curcumin can improve PA-induced PINK1/Parkin-mediated mitophagy.

### Curcumin improves insulin sensitivity via regulating PINK1

**Fig. 4:**
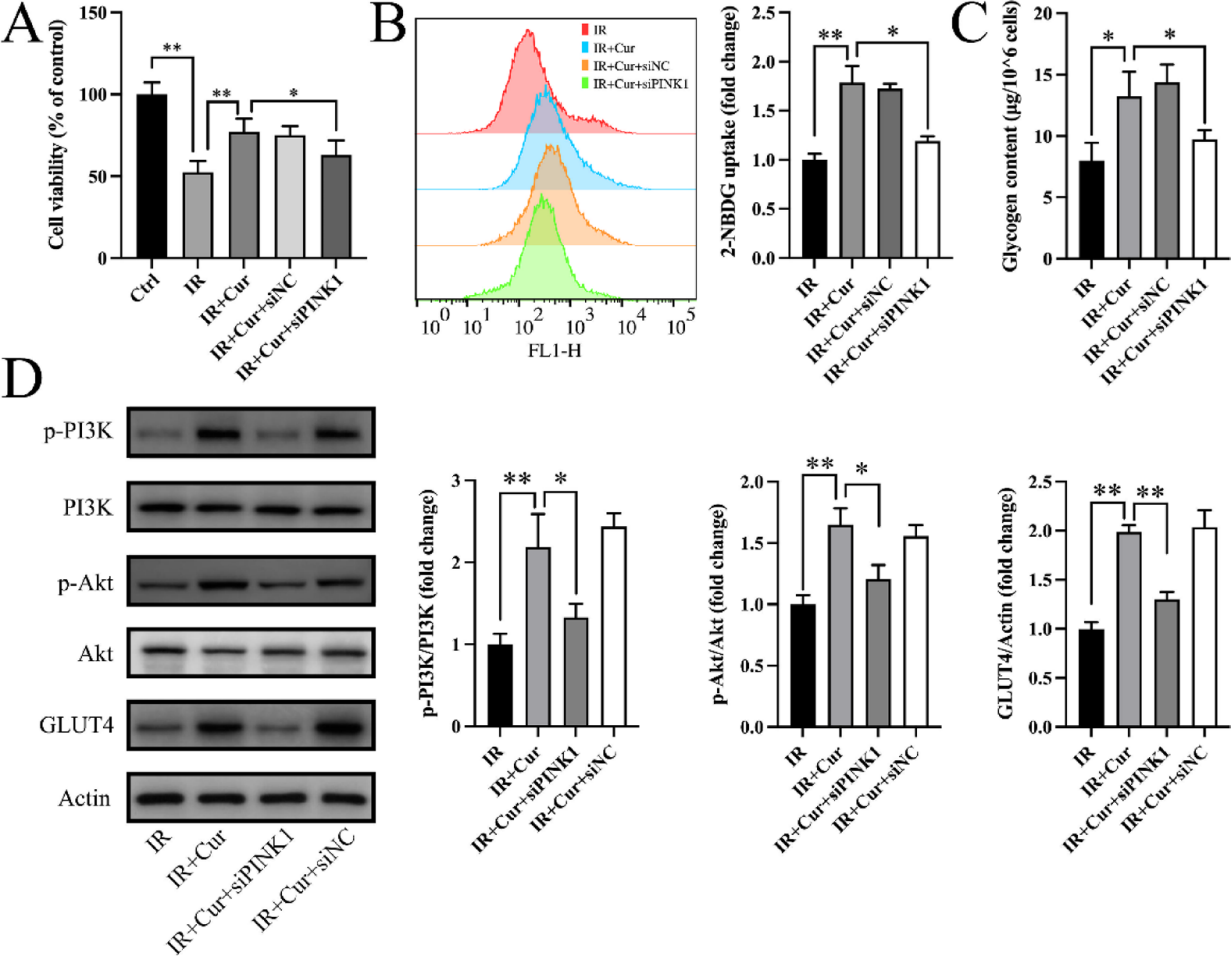
Inhibition of PINK1 partially reversed effects of curcumin on insulin resistance. (**A**) Cell viability assay (*n* = 6). (**B**) 2-NBDG uptake (*n* = 3). (**C**) Intracellular glycogen content (*n* = 3). (D) PI3K/Akt phosphorylation and GLUT4 expression (*n* = 3). (Tukey’s HSD, * *P* < 0.05 ** *P* < 0.01)

To investigate the involvement of PINK1 signaling in curcumin-mediated improvement of insulin resistance, small interfering RNA targeting PINK1 (siPINK1) and a negative control (siNC) were transfected into HepG2 cells. The findings indicated that curcumin increased cell viability (147 ± 15.2% of IR group, Fig. 4A), enhanced 2-NBDG uptake (179 ± 16.9% of IR group, Fig. 4B), and elevated glycogen content (166 ± 24.9% of IR group, Fig. 4C). PINK1 knockdown partially reversed these beneficial effects (Fig. 4A-C). Western blot revealed that curcumin promoted GLUT4 expression and phosphorylation of PI3K/Akt, whereas siPINK1 partially blocked these effects (Fig. 4D). These findings suggest that curcumin ameliorates insulin resistance primarily through regulating PINK1.

### Curcumin improves insulin sensitivity via activating PINK1-dependent mitophagy

**Fig. 5:**
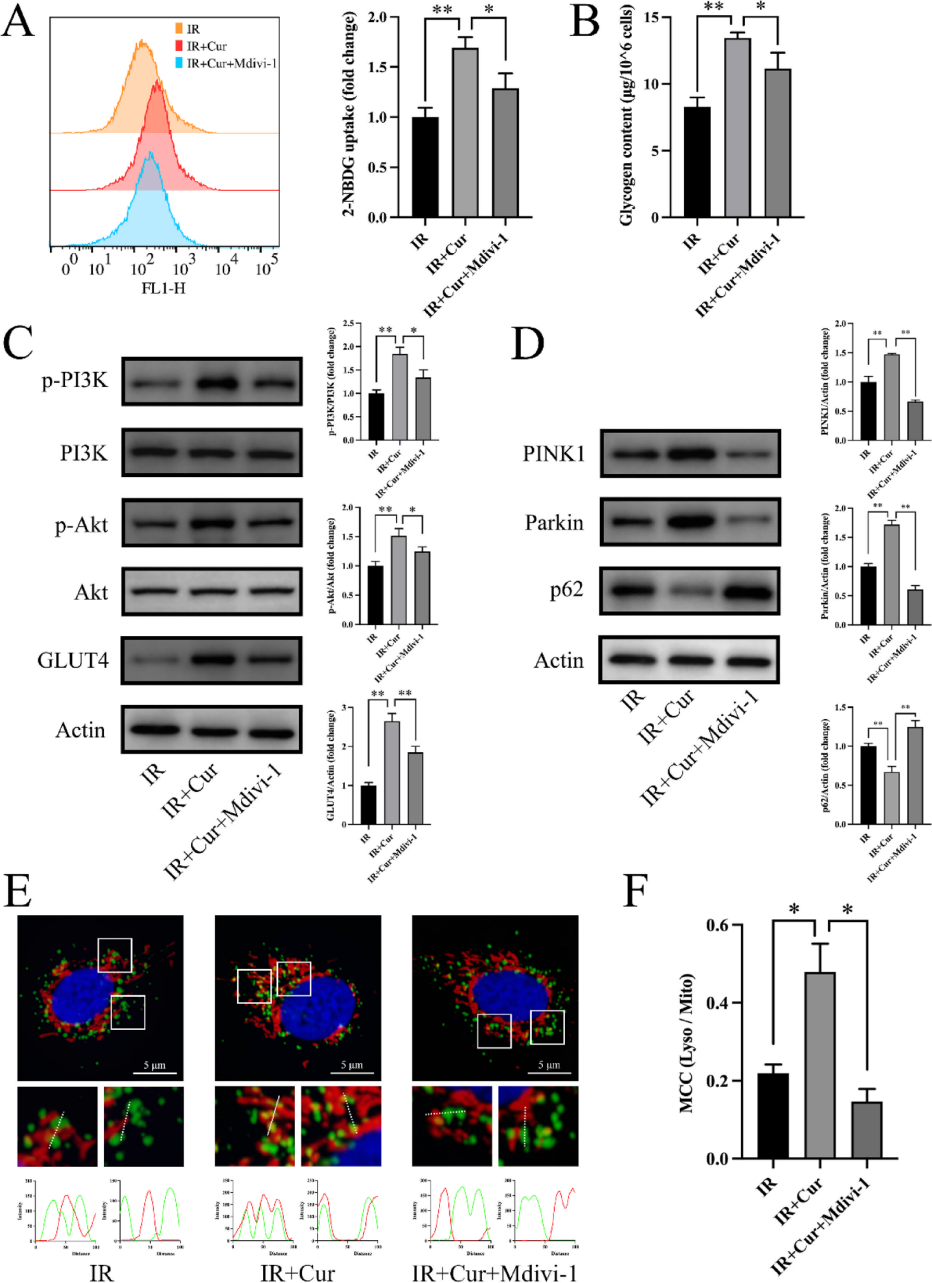
Mdivi-1 partially reversed the impact of curcumin on insulin resistance in HepG2 cells (*n* = 3). (**A**) 2-NBDG uptake. (**B**) Intracellular glycogen content. (**C**) PI3K/Akt phosphorylation and GLUT4 expression. (**D**) Protein expression levels of PINK1/Parkin/p62. (**E**) Colocalization of mitochondria and lysosomes. (F) Manders’ colocalization coefficient of lysosomes and mitochondria. (Tukey’s HSD, * *P* < 0.05, ** *P* < 0.01)

To further explore how curcumin improves insulin resistance, we used the mitophagy inhibitor Mdivi-1 to block mitophagy. We found that curcumin treatment increased 2-NBDG uptake (169 ± 10.6% of IR group, Fig. 5A) and glycogen content (162 ± 4.99% of IR group, Fig. 5B) in PA-treated HepG2 cells, enhanced phosphorylation of PI3K/Akt signaling pathway, and promoted GLUT4 expression (Fig. 5C). However, these effects of curcumin were partially reversed by Mdivi-1 (Fig. 5A-C). We next examined proteins related to the PINK1 signaling pathway by Western blot. Curcumin increased expression of PINK1/Parkin and reduced p62, these effects were markedly reversed by Mdivi-1 (Fig. 5D). Immunofluorescence analysis showed that curcumin increased the overlap of mitochondrial and lysosomal fluorescence signals (Fig. 5E) and the Manders’ colocalization coefficient (219 ± 32.9% of Ctrl group, Fig. 5F) in insulin resistant HepG2 cells, indicating enhanced mitophagy. This effect was also significantly reversed by Mdivi-1 (30.7 ± 6.63% of IR + Cur group, Fig. 5E-F). These findings indicate that in HepG2 cells, curcumin enhances glucose uptake and utilization and thereby improves PA-induced insulin resistance, at least in part by activating PINK1/Parkin-mediated mitophagy.

## Discussion

Curcumin is a plant derived polyphenol from *Curcuma longa* with antioxidant and anti-inflammatory actions^22^. Prior work links curcumin to protection against obesity, T2DM, and non-alcoholic fatty liver disease (NAFLD) through modulation of inflammatory cytokines, redox balance, and insulin signaling^22–24^, yet the mechanism by which it alleviates insulin resistance remains incompletely defined. In our study, curcumin markedly ameliorated PA-induced insulin resistance in HepG2 cells. The benefit aligns with activation of PINK1-dependent mitophagy. These findings provide evidence for pharmacological effects of curcumin in improving insulin resistance.

Insulin resistance refers to a weakened response of insulin target tissues, resulting in impaired glucose uptake and utilization. It is a common pathological basis for many metabolic diseases, including T2DM and NAFLD^25^. Studies have focused on the effects of natural compounds on regulating glucose metabolism and reducing chronic inflammation, finding that they have advantages of multi-target action and low toxicity in improving insulin resistance. Bioactive constituents from plants, including flavonoids and polyphenols, have been proven to improve target tissue sensitivity to insulin via activating PI3K/Akt signaling, enhancing GLUT4 expression and translocation, improving mitochondrial function and inhibiting inflammation^26–28^. Consistent with these findings, we observed that curcumin ameliorates PA-induced insulin resistance in HepG2 cells, as manifested by promoting glucose uptake and increasing glycogen content. Western blot analysis further confirmed that curcumin increased phosphorylation of PI3K/Akt and enhanced GLUT4 expression. HepG2 cells possess essential glucose and lipid metabolism functions of human hepatocytes, including insulin receptor, GLUTs, and major enzymes that participate in glycogen synthesis and gluconeogenesis^29^. Many studies have induced insulin resistance in HepG2 cells to evaluate the antidiabetic effects of various compounds in hepatocytes^30,31^. However, HepG2 cells are immortalized hepatocellular carcinoma cells, and their metabolism differs from that of primary hepatocytes^32^. Their glycolytic activity is high and they are not polarized like normal liver cells^33^. Our results reveal that curcumin has the potential to alleviate insulin resistance at the cellular level. To verify its pharmacological effects, it will be required to further test it with primary hepatocytes or animal models.

Mitophagy is an important mechanism for removing damaged or dysfunctional mitochondria through selective autophagy^16^. In the process, damaged mitochondria are selectively marked and wrapped by autophagosomes, and cleared after lysosome fusion to limit overproduction of ROS^16,34^. Studies have found that impaired mitophagy is associated with several metabolic disorders, including T2DM and NAFLD. If this quality control pathway is absent, dysfunctional mitochondria will accumulate, resulting in reduced ATP production and enhanced ROS production, ultimately aggravating insulin resistance^34–36^. Studies have reported that polyphenolic compounds such as resveratrol and polydatin can reduce hepatic lipid accumulation and inflammation by activating mitophagy, thus improving NAFLD^37–39^. PINK1/Parkin pathway is important for regulating mitophagy. When mitochondrial membrane potential drops, PINK1 accumulates and recruits Parkin to the mitochondrial outer membrane. This promotes recognition of autophagy receptors and clearance of damaged mitochondria^40^. Hepatocyte specific deletion of Parkin impairs mitochondrial respiration and aggravates NAFLD and insulin resistance. Activation of this pathway can reverse these conditions^41^. In our study, PA suppressed PINK1/Parkin protein expression and reduced mitochondria-lysosome colocalization, indicating impaired mitophagy under insulin resistant conditions. Curcumin restored PINK1/Parkin-dependent mitophagy and improved mitochondrial membrane potential, ATP production and OCR, indicating enhanced mitochondrial function. However, the interaction between PINK1/Parkin and insulin resistance remains unclear. In this study, we used siRNA to silence the PINK1 and found that the impact of curcumin on insulin resistance and mitochondrial dysfunction was partially reversed. Similarly, inhibition of mitophagy by Mdivi-1 also blocked these effects of curcumin, suggesting that activation of PINK1/Parkin-dependent mitophagy contributes, at least in part, to curcumin’s ability to improve insulin resistance. All these observations confirm that mitochondrial quality control plays a vital role in metabolic control and that its pharmacological regulation can have therapeutic effects independent of antioxidant activity.

Our study found that Mdivi-1 worsens insulin resistance by inhibiting mitophagy. However, we should note that Mdivi-1 has been shown to improve insulin sensitivity in other cell models. For example, Jheng et al. reported that Mdivi-1 reduces PA-induced mitochondrial fragmentation, membrane potential loss and insulin resistance by inhibiting dynamin-related protein 1 (Drp1)^42^. This seemingly contradictory finding shows that mitochondria quality control is strictly linked to cell and disease specific processes. In this study, PA caused accumulation of damaged mitochondria in HepG2 cells, and mitophagy facilitated by PINK1 was an adaptive response to achieve metabolic homeostasis. Inhibiting this pathway by Mdivi-1 prevented clearance of dysfunctional mitochondria, eventually increasing insulin resistance.

Interestingly, both the mitophagy inhibitor Mdivi-1 and siPINK1 can only partially reverse the effects of curcumin. This suggests that additional mechanisms contribute to its insulin-sensitizing effect. Curcumin is known to downregulate proinflammatory cytokines and inhibit inflammatory signaling including nuclear factor kappa B (NF-κB), mitogen-activated protein kinase (MAPK) and JNK, thus reducing chronic inflammation and improving insulin responses in target tissues^22,43^. In addition, excessive ROS production and imbalance of antioxidant defense are all factors in the development of insulin resistance. Curcumin has been reported to activate the Nrf2 pathway, increase the efficacy of antioxidant enzymes and reduce lipid peroxidation products, thereby alleviating oxidative damage^44^. Therefore, the mitophagy pathway may serve as an important, but not the only mechanism by which curcumin improves insulin resistance.

## Conclusion

In summary, impaired mitophagy contributes to PA-induced insulin resistance. Curcumin improves cellular glucose handling and reverses insulin sensitivity by reactivating mitophagy and repairing mitochondrial function. This effect is mediated, at least partially, by activation of the PINK1/Parkin pathway. These findings highlight PINK1/Parkin-mediated mitophagy as a therapeutic target for insulin resistance. Further studies should verify these effects of curcumin in vivo and evaluate its ability to ameliorate insulin resistance in skeletal muscle and adipose tissue.

## Supplementary Information

Below is the link to the electronic supplementary material.

Supplementary Material 1

## Data Availability

The data supporting the results of this study are not publicly available but can be obtained from the corresponding author upon reasonable request.

## References

[1] Collaborators, G. B. D. D. Global, regional, and national burden of diabetes from 1990 to 2021, with projections of prevalence to 2050: a systematic analysis for the Global Burden of Disease Study 2021[J]. *Lancet* **402** (10397), 203–234 (2023). 37356446 10.1016/S0140-6736(23)01301-6 PMC10364581

[2] Abdulghani, M. F. & Al-Fayyadh, S. Natural products for managing metabolic syndrome: a scoping review[J]. *Front. Pharmacol.* **15**, 1366946 (2024). 38746011 10.3389/fphar.2024.1366946 PMC11091304

[3] Martiniakova, M. et al. Protective Role of Dietary Polyphenols in the Management and Treatment of Type 2 Diabetes Mellitus[J]. *Nutrients*, **17**(2). (2025). 10.3390/nu17020275 PMC11767469 39861406

[4] Kumar, S. et al. Phloretin and phlorizin mitigates inflammatory stress and alleviate adipose and hepatic insulin resistance by abrogating PPARgamma S273-Cdk5 interaction in type 2 diabetic mice[J]. *Life Sci.* **322**, 121668 (2023). 37023949 10.1016/j.lfs.2023.121668

[5] Zhu, Y. et al. Ginsenoside Rg5 Improves Insulin Resistance and Mitochondrial Biogenesis of Liver via Regulation of the Sirt1/PGC-1alpha Signaling Pathway in db/db Mice[J]. *J. Agric. Food Chem.* **69** (30), 8428–8439 (2021). 34309383 10.1021/acs.jafc.1c02476

[6] Veluthakal, R. et al. Mitochondrial Dysfunction, Oxidative Stress, and Inter-Organ Miscommunications in T2D Progression[J]. *Int. J. Mol. Sci.*, **25**(3). (2024). 10.3390/ijms25031504 PMC10855860 38338783

[7] Zheng, Z. G. et al. Ergosterol alleviates hepatic steatosis and insulin resistance via promoting fatty acid beta-oxidation by activating mitochondrial ACSL1[J]. *Cell. Rep.* **44** (1), 115203 (2025). 39799570 10.1016/j.celrep.2024.115203

[8] Chen, Y. et al. Berberine protects mice against type 2 diabetes by promoting PPARgamma-FGF21-GLUT2-regulated insulin sensitivity and glucose/lipid homeostasis[J]. *Biochem. Pharmacol.* **218**, 115928 (2023). 37979703 10.1016/j.bcp.2023.115928

[9] Du, F. et al. Resveratrol Improves Liver Steatosis and Insulin Resistance in Non-alcoholic Fatty Liver Disease in Association With the Gut Microbiota[J]. *Front. Microbiol.* **12**, 611323 (2021). 33708180 10.3389/fmicb.2021.611323 PMC7942199

[10] Li, L. et al. *Metformin improves insulin resistance, liver healthy and abnormal hepatic glucolipid metabolism via IR/PI(3)K/AKT pathway in Ctenopharyngodon idella fed a high-carbohydrate diet[J]:* (*Comp Biochem Physiol C Toxicol Pharmacol* 283, 109976.(2024). 10.1016/j.cbpc.2024.109976 38987002

[11] Hao, M. et al. Pharmacological effects, formulations, and clinical research progress of curcumin[J]. *Front. Pharmacol.* **16**, 1509045 (2025). 40166470 10.3389/fphar.2025.1509045 PMC11955698

[12] Farzaei, M. H. et al. Curcumin in Liver Diseases: A Systematic Review of the Cellular Mechanisms of Oxidative Stress and Clinical Perspective[J]. *Nutrients*, **10**(7). (2018). 10.3390/nu10070855 PMC6073929 29966389

[13] Zheng, X. et al. The effect of curcumin supplementation on glycemic indices in adults: A meta-analysis of meta-analyses[J]. *Prostaglandins Other Lipid Mediat*. **175**, 106908 (2024). 39270815 10.1016/j.prostaglandins.2024.106908

[14] Chen, M., Yao, J. & Liu, Z. Curcumin ameliorates high-glucose-induced lipid accumulation in HepG2 cells via AMPK activation and mTOR suppression[J]. *Biochem. Biophys. Res. Commun.* **790**, 152879 (2025). 41172806 10.1016/j.bbrc.2025.152879

[15] Jin, Z. et al. Curcumin exerts chondroprotective effects against osteoarthritis by promoting AMPK/PINK1/Parkin-mediated mitophagy[J]. *Biomed. Pharmacother*. **151**, 113092 (2022). 35550528 10.1016/j.biopha.2022.113092

[16] Picca, A. et al. Mitophagy in human health, ageing and disease[J]. *Nat. Metab.* **5** (12), 2047–2061 (2023). 38036770 10.1038/s42255-023-00930-8 PMC12159423

[17] Chen, Y. et al. Mitochondrial quality control in diabetes mellitus and complications: molecular mechanisms and therapeutic strategies[J]. *Cell. Death Dis.* **16** (1), 652 (2025). 40866350 10.1038/s41419-025-07936-y PMC12391366

[18] Rustamov, N. et al. Korean Red Ginseng Improves Oxidative Stress-Induced Hepatic Insulin Resistance via Enhancing Mitophagy[J]. Foods, 13(**13**). (2024). 10.3390/foods13132137 PMC11241528 38998642

[19] Geng, S. et al. Curcumin suppresses JNK pathway to attenuate BPA-induced insulin resistance in LO2 cells[J]. *Biomed. Pharmacother*. **97**, 1538–1543 (2018). 29793316 10.1016/j.biopha.2017.11.069

[20] Sarmiento-Ortega, V. E. et al. Curcumin Treatment Ameliorates Hepatic Insulin Resistance Induced by Sub-chronic Oral Exposure to Cadmium LOAEL Dose via NF-kappaB and Nrf2 Pathways[J]. *Biol. Trace Elem. Res.* **203** (4), 2382–2393 (2025). 39103711 10.1007/s12011-024-04314-1 PMC11919948

[21] Nie, J. et al. Caffeic Acid Phenethyl Ester (Propolis Extract) Ameliorates Insulin Resistance by Inhibiting JNK and NF-kappaB Inflammatory Pathways in Diabetic Mice and HepG2 Cell Models[J]. *J. Agric. Food Chem.* **65** (41), 9041–9053 (2017). 28799756 10.1021/acs.jafc.7b02880

[22] Hussain, Y. et al. How Curcumin Targets Inflammatory Mediators in Diabetes: Therapeutic Insights and Possible Solutions[J]. *Molecules*, **27**(13). (2022). 10.3390/molecules27134058 PMC9268477 35807304

[23] Qiu, L. et al. Effects of dietary polyphenol curcumin supplementation on metabolic, inflammatory, and oxidative stress indices in patients with metabolic syndrome: a systematic review and meta-analysis of randomized controlled trials[J]. *Front. Endocrinol. (Lausanne)*. **14**, 1216708 (2023). 37522129 10.3389/fendo.2023.1216708 PMC10376715

[24] Li, P. et al. Curcumin metabolites contribute to the effect of curcumin on ameliorating insulin sensitivity in high-glucose-induced insulin-resistant HepG2 cells[J]. *J. Ethnopharmacol.* **259**, 113015 (2020). 32464315 10.1016/j.jep.2020.113015

[25] Tanase, D. M. et al. The Intricate Relationship between Type 2 Diabetes Mellitus (T2DM), Insulin Resistance (IR), and Nonalcoholic Fatty Liver Disease (NAFLD)[J]. J Diabetes Res, 2020:3920196. (2020). 10.1155/2020/3920196 PMC7424491 32832560

[26] Izbicka, E. & Streeper, R. T. Mitigation of Insulin Resistance by Natural Products from a New Class of Molecules. *Membrane-Active Immunomodulators[J] Pharmaceuticals (Basel)*, **16**(7). (2023). 10.3390/ph16070913 PMC10386479 37513825

[27] Ansari, P. et al. Plant-Based Diets and Phytochemicals in the Management of Diabetes Mellitus and Prevention of Its Complications: A Review[J]. *Nutrients*, **16**(21). (2024). 10.3390/nu16213709 PMC11547802 39519546

[28] Niazpour, F. & Meshkani, R. Unlocking the Therapeutic Potential of Autophagy Modulation by Natural Products in Tackling Non-Alcoholic Fatty Liver Disease[J]. *Phytother Res.* **39** (5), 2357–2373 (2025). 40184168 10.1002/ptr.8463

[29] Bonanini, F. et al. A comparison between different human hepatocyte models reveals profound differences in net glucose production, lipid composition and metabolism in vitro[J]. *Exp. Cell. Res.* **437** (1), 114008 (2024). 38499143 10.1016/j.yexcr.2024.114008

[30] Zhang, S. et al. Activation of NRF2 by epiberberine improves oxidative stress and insulin resistance in T2DM mice and IR-HepG2 cells in an AMPK dependent manner[J]. *J. Ethnopharmacol.* **327**, 117931 (2024). 38382657 10.1016/j.jep.2024.117931

[31] Song, L., Li, Y. & Xu, M. Exogenous Nucleotides Ameliorate Insulin Resistance Induced by Palmitic Acid in HepG2 Cells through the IRS-1/AKT/FOXO1 Pathways[J]. *Nutrients*, **16**(12). (2024). 10.3390/nu16121801 PMC11206901 38931156

[32] Huggett, Z. J. et al. A Comparison of Primary Human Hepatocytes and Hepatoma Cell Lines to Model the Effects of Fatty Acids, Fructose and Glucose on Liver Cell Lipid Accumulation[J]. *Nutrients*, **15**(1). (2022). 10.3390/nu15010040 PMC9824391 36615698

[33] Arzumanian, V., Pyatnitskiy, M. & Poverennaya, E. Comparative Transcriptomic Analysis of Three Common Liver Cell Lines[J]. *Int. J. Mol. Sci.*, **24**(10). (2023). 10.3390/ijms24108791 PMC10218629 37240140

[34] Ning, P. et al. Mitophagy: A potential therapeutic target for insulin resistance[J]. *Front. Physiol.* **13**, 957968 (2022). 36082218 10.3389/fphys.2022.957968 PMC9445132

[35] Su, Z. et al. Phytochemicals: Targeting Mitophagy to Treat Metabolic Disorders[J]. *Front. Cell. Dev. Biol.* **9**, 686820 (2021). 34414181 10.3389/fcell.2021.686820 PMC8369426

[36] Undamatla, R. et al. Reduced mitophagy is an early feature of NAFLD and liver-specific PARKIN knockout hastens the onset of steatosis, inflammation and fibrosis[J]. *Sci. Rep.* **13** (1), 7575 (2023). 37165006 10.1038/s41598-023-34710-x PMC10172344

[37] Liu, Y., Zhang, M. & Wang, Y. Induction of Autophagy as a Therapeutic Breakthrough for NAFLD: Current Evidence and Perspectives[J]. *Biology (Basel)*, **14**(8). (2025). 10.3390/biology14080989 PMC12383869 40906106

[38] He, J. et al. Polydatin: a potential NAFLD therapeutic drug that regulates mitochondrial autophagy through SIRT3-FOXO3-BNIP3 and PINK1-PRKN mechanisms - a network pharmacology and experimental investigation[J]. *Chem. Biol. Interact.* **398**, 111110 (2024). 38876248 10.1016/j.cbi.2024.111110

[39] He, X. et al. Integrative evidence construction for resveratrol treatment of nonalcoholic fatty liver disease: preclinical and clinical meta-analyses[J]. *Front. Pharmacol.* **14**, 1230783 (2023). 37767399 10.3389/fphar.2023.1230783 PMC10520779

[40] Killackey, S. A., Philpott, D. J. & Girardin, S. E. Mitophagy pathways in health and disease[J]. *J. Cell. Biol.*, **219**(11). (2020). 10.1083/jcb.202004029 PMC7594502 32926082

[41] Huang, Y. et al. Mitophagy as a pivotal axis in non–alcoholic fatty liver disease: From pathogenic mechanisms to therapeutic strategies (Review)[J]. *Mol. Med. Rep.*, **32**(5). (2025). 10.3892/mmr.2025.13664 PMC12409231 40878939

[42] Jheng, H. F. et al. Mitochondrial fission contributes to mitochondrial dysfunction and insulin resistance in skeletal muscle[J]. *Mol. Cell. Biol.* **32** (2), 309–319 (2012). 22083962 10.1128/MCB.05603-11 PMC3255771

[43] Peng, Y. et al. Anti-Inflammatory Effects of Curcumin in the Inflammatory Diseases: Status, Limitations and Countermeasures[J]. *Drug Des. Devel Ther.* **15**, 4503–4525 (2021). 34754179 10.2147/DDDT.S327378 PMC8572027

[44] Cui, J. et al. Research progress on the mechanism of curcumin anti-oxidative stress based on signaling pathway[J]. *Front. Pharmacol.* **16**, 1548073 (2025). 40260389 10.3389/fphar.2025.1548073 PMC12009910

