# Supplementary material for: Curcumin ameliorates hepatic insulin resistance by activating PINK1/Parkin-mediated mitophagy
Source: Sci Rep. 2026 Apr 8;16:16667. doi: 10.1038/s41598-026-47924-6 (PMC13219394; doi:10.1038/s41598-026-47924-6)

Fig. 1C repeat1

p-PI3K (85 kDa)

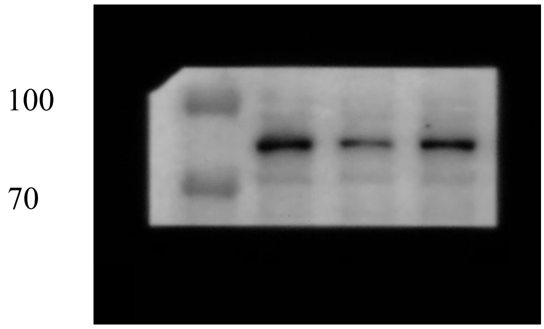

p-Akt (60 kDa)

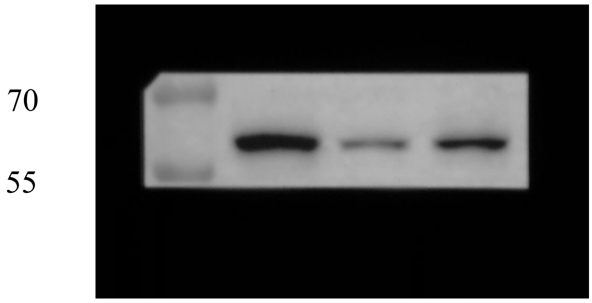

PI3K (85 kDa)

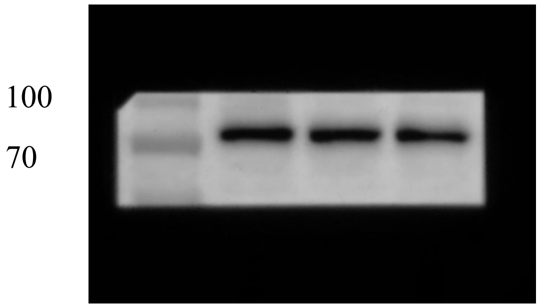

Akt (60 kDa)

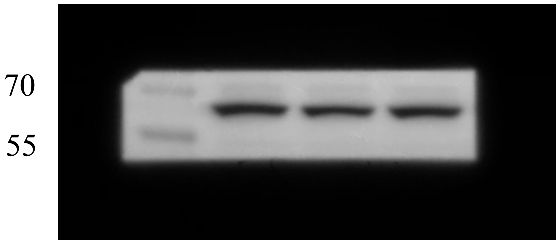

GLUT4 (53 kDa)

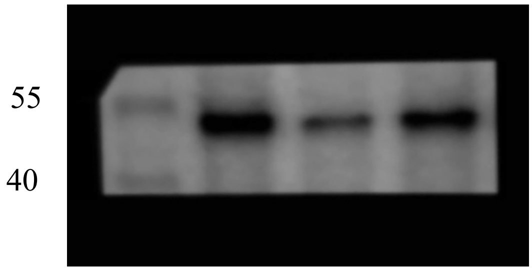

Actin (42 kDa)

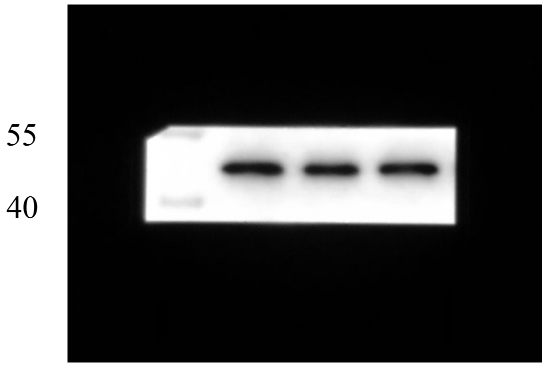

Fig. 1C repeat2

p-PI3K (85 kDa)

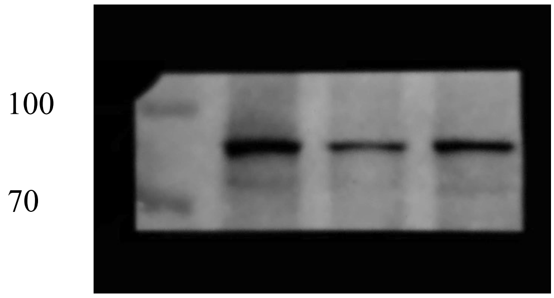

p-Akt (60 kDa)

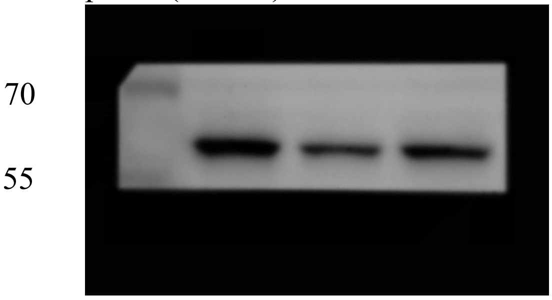

PI3K (85 kDa)

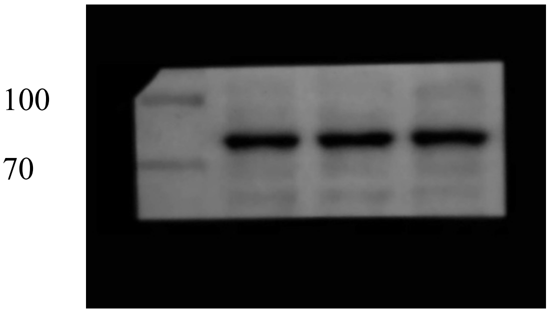

Akt (60 kDa)

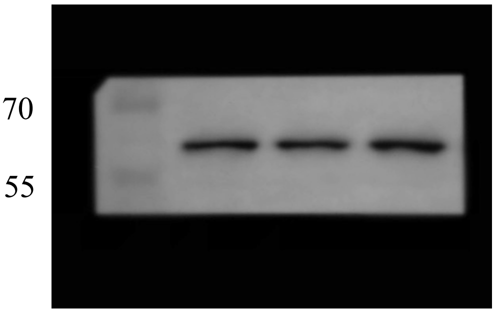

GLUT4 (53 kDa)

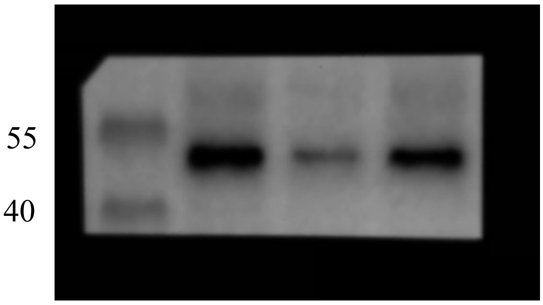

Actin (42 kDa)

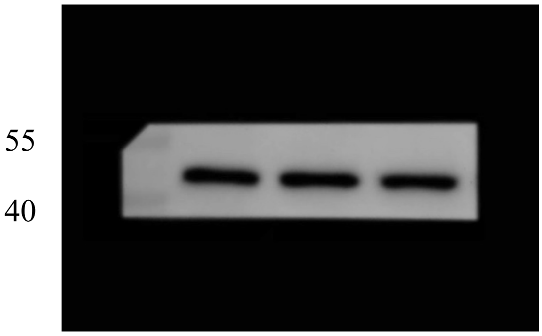

Fig. 1C repeat3

p-PI3K (85 kDa)

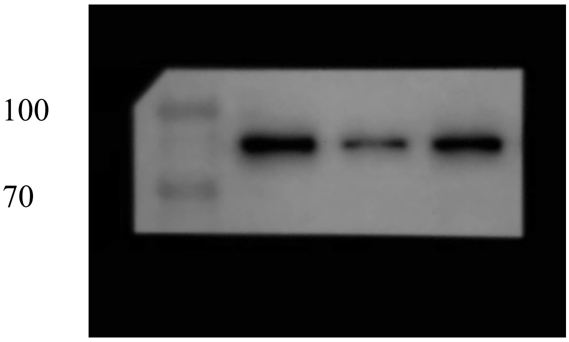

p-Akt (60 kDa)

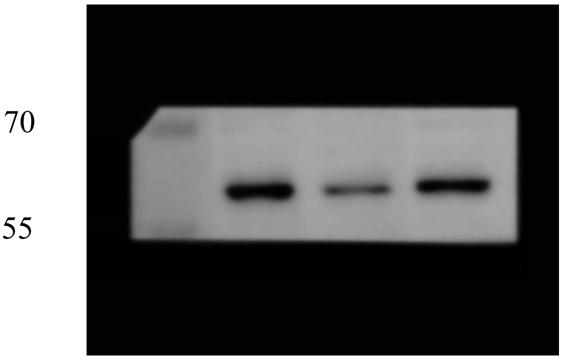

PI3K (85 kDa)

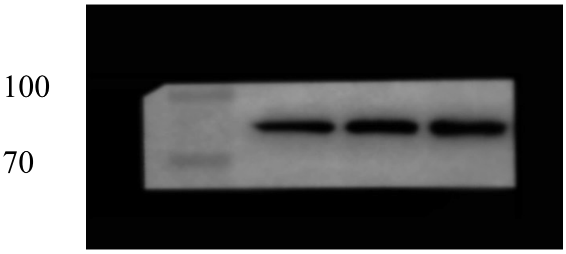

Akt (60 kDa)

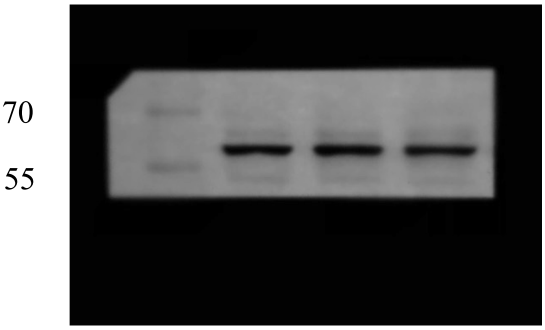

GLUT4 (53 kDa)

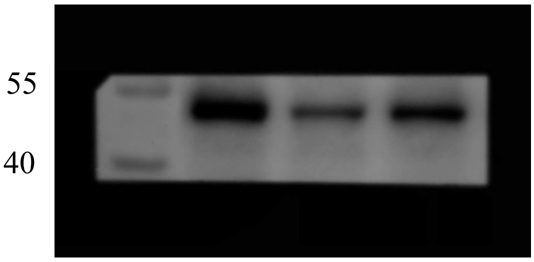

Actin (42 kDa)

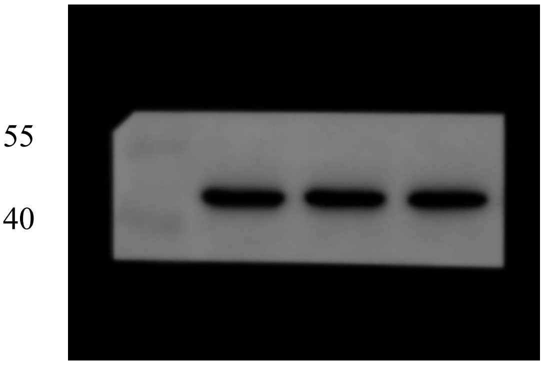

Fig. 3A repeat1

PINK1 (63 kDa)

70
55

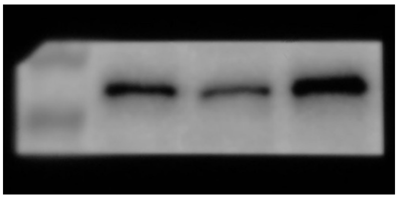

Parkin (50 kDa)

55
40

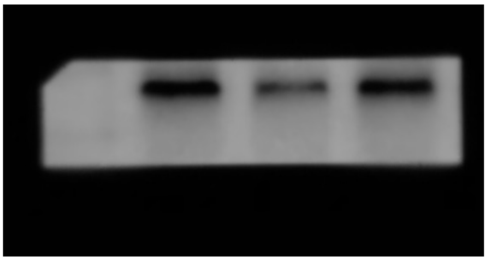

p62 (62 kDa)

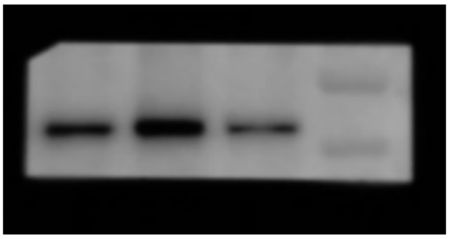

70
55

Actin (42 kDa)

55
40

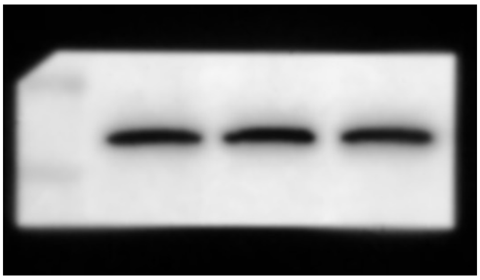

Fig. 3A repeat2

PINK1 (63 kDa)

70
55

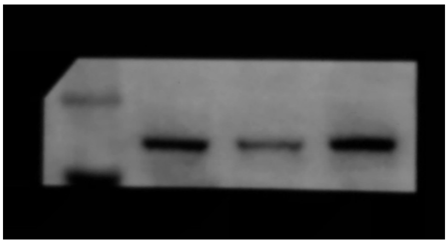

Parkin (50 kDa)

55
40

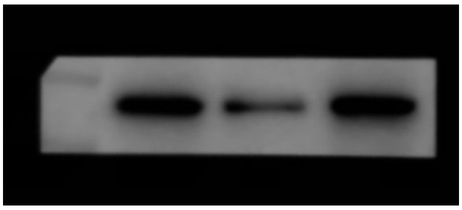

p62 (62 kDa)

70
55

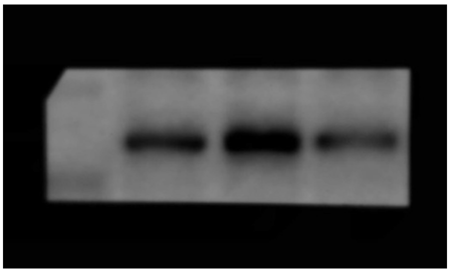

Actin (42 kDa)

55
40

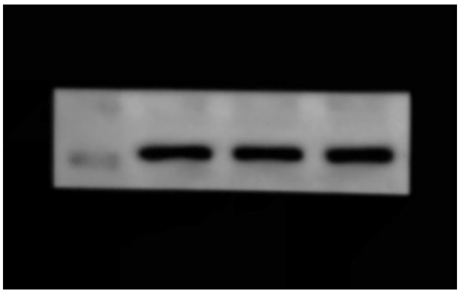

Fig. 3A repeat3

PINK1 (63 kDa)

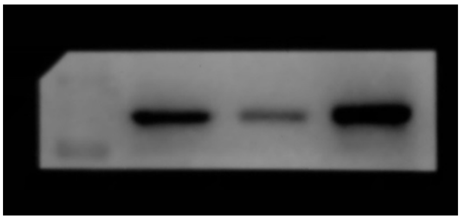

Parkin (50 kDa)

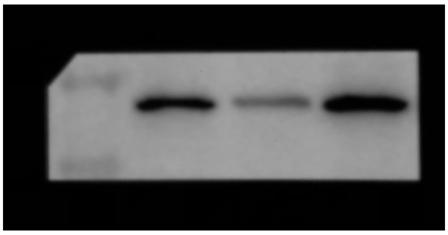

p62 (62 kDa)

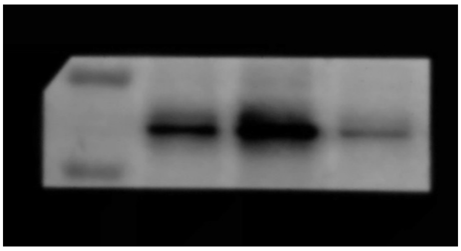

Actin (42 kDa)

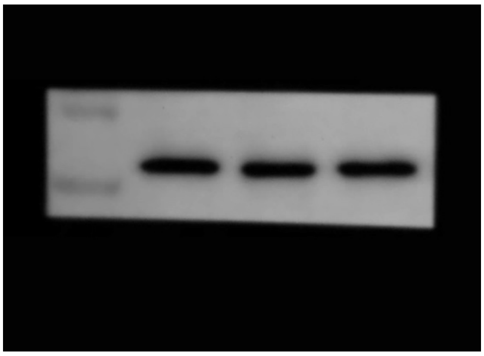

Fig. 4D repeat1

p-PI3K (85 kDa)

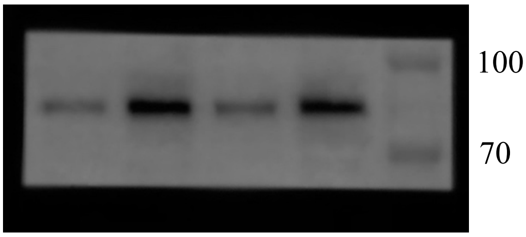

PI3K (85 kDa)

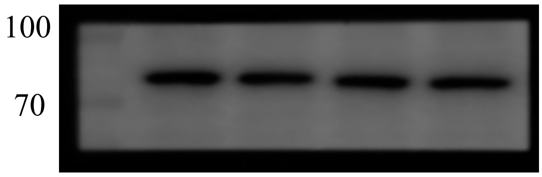

p-Akt (60 kDa)

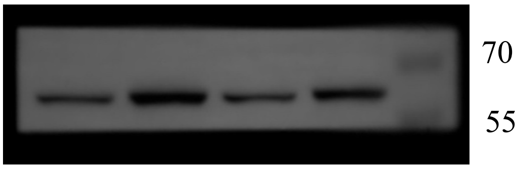

Akt (60 kDa)

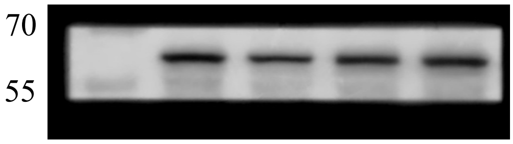

GLUT4 (53 kDa)

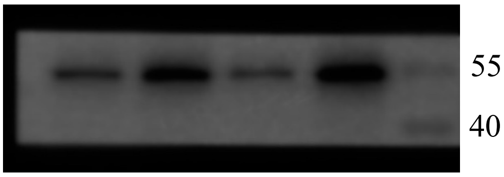

Actin (42 kDa)

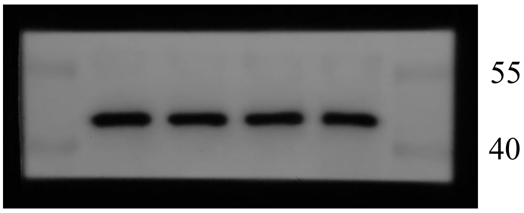

Fig. 4D repeat2

p-PI3K (85 kDa)

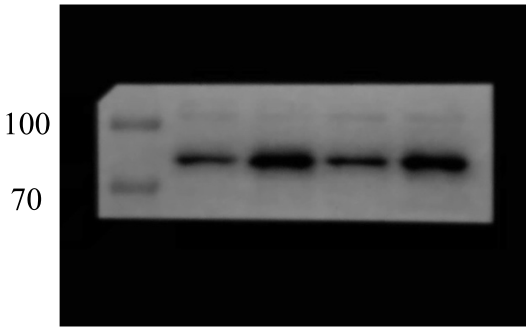

PI3K (85 kDa)

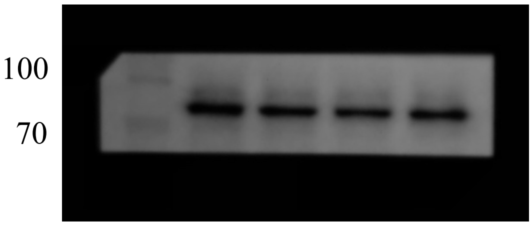

p-Akt (60 kDa)

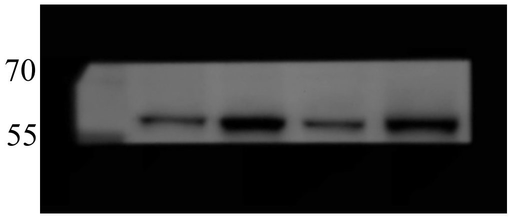

Akt (60 kDa)

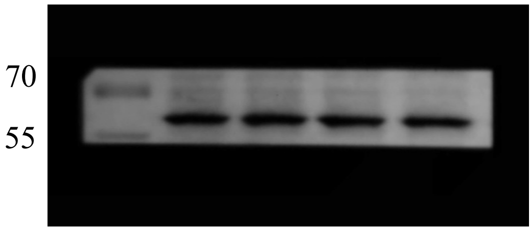

GLUT4 (53 kDa)

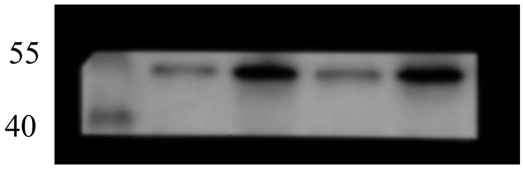

Actin (42 kDa)

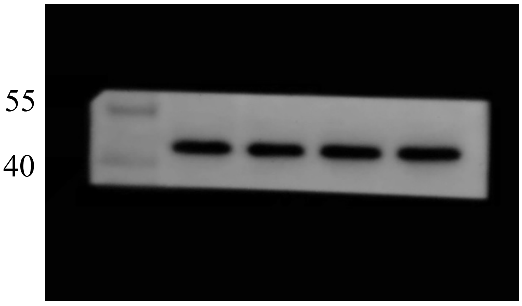

Fig. 4D repeat3

p-PI3K (85 kDa)

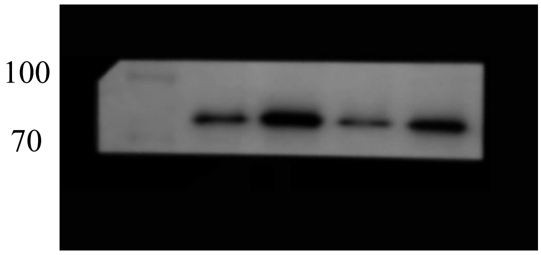

PI3K (85 kDa)

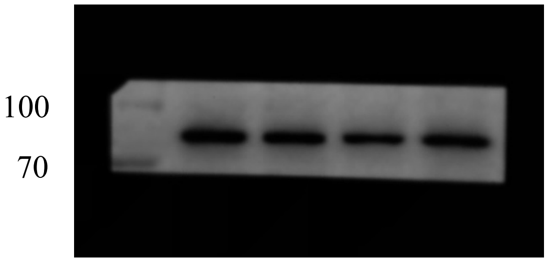

p-Akt (60 kDa)

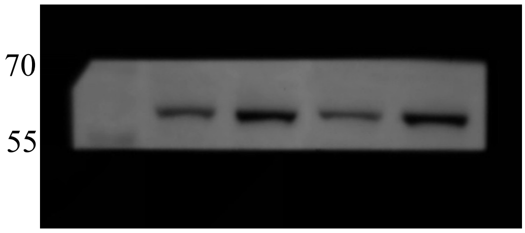

Akt (60 kDa)

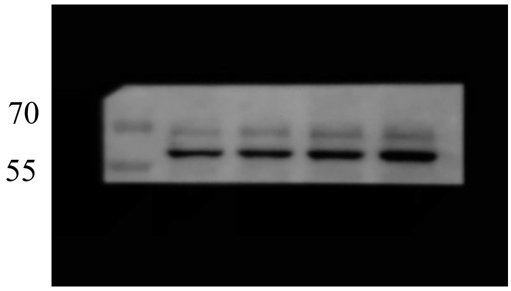

GLUT4 (53 kDa)

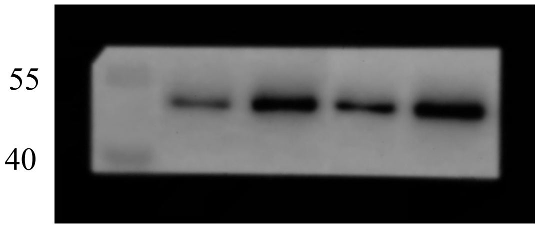

Actin (42 kDa)

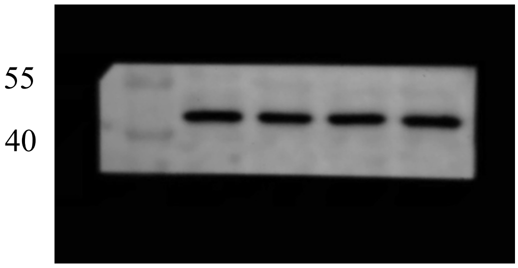

Fig. 5C repeat1

p-PI3K (85 kDa)

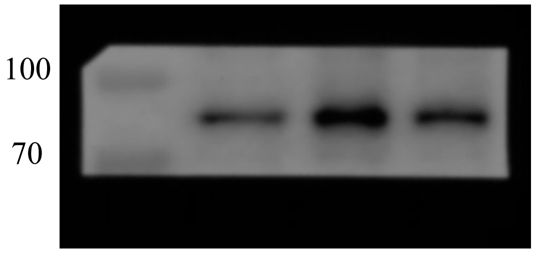

PI3K (85 kDa)

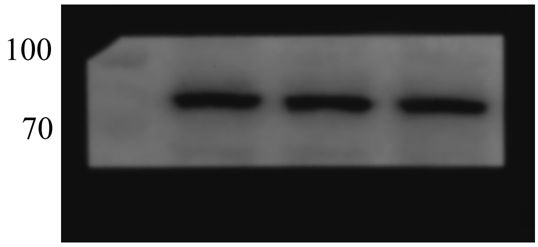

p-Akt (60 kDa)

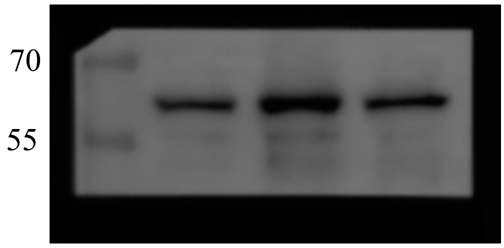

Akt (60 kDa)

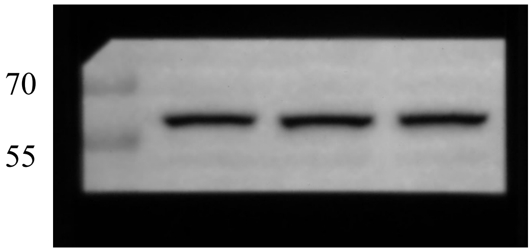

GLUT4 (53 kDa)

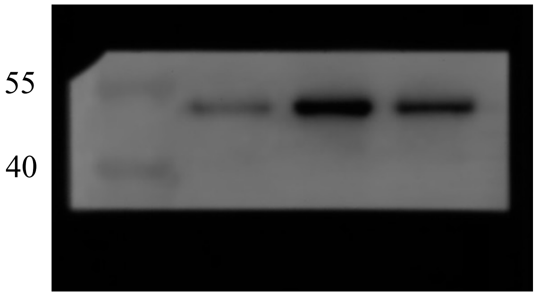

Actin (42 kDa)

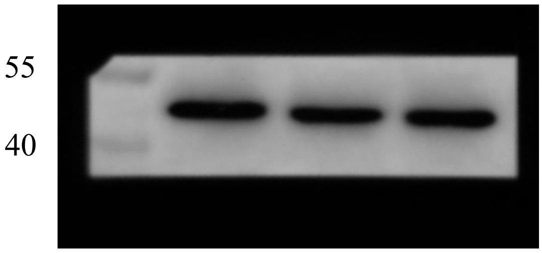

Fig. 5C repeat2

p-PI3K (85 kDa)

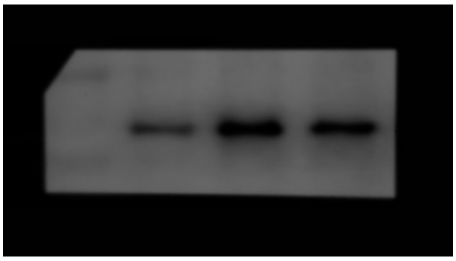
100
70

PI3K (85 kDa)

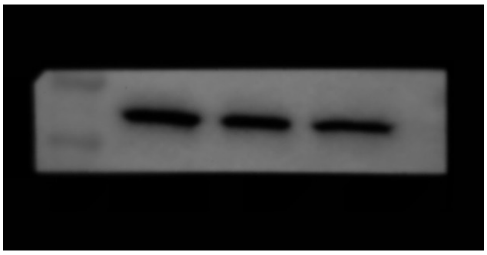
100
70

p-Akt (60 kDa)

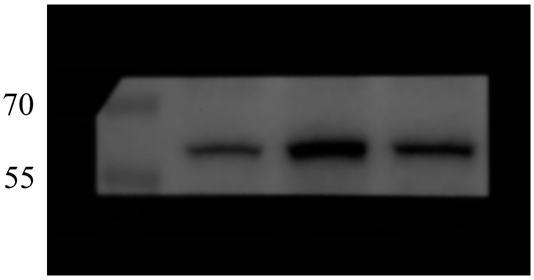
70
55

Akt (60 kDa)

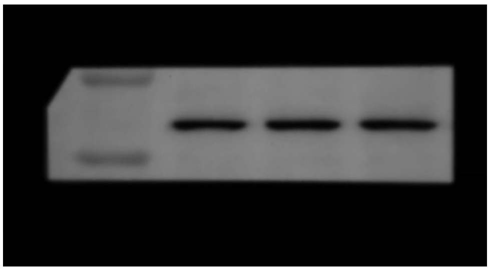
70
55

GLUT4 (53 kDa)

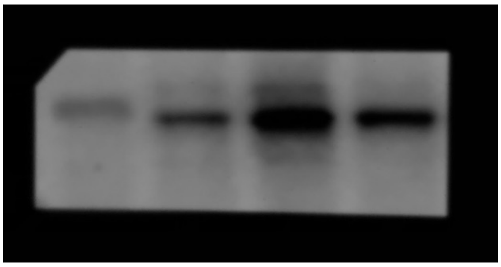
55
40

Actin (42 kDa)

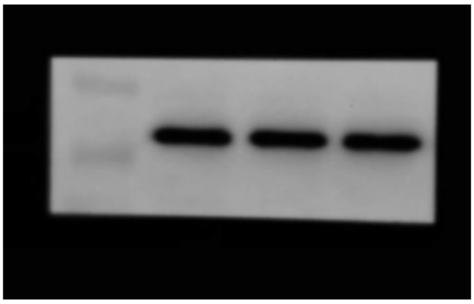
55
40

Fig. 5C repeat3

p-PI3K (85 kDa)

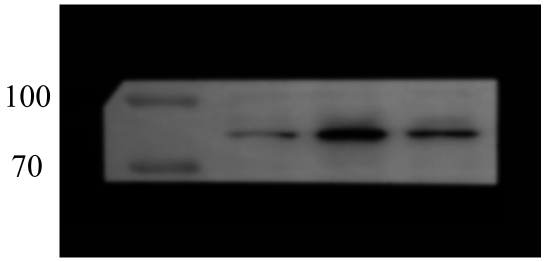

PI3K (85 kDa)

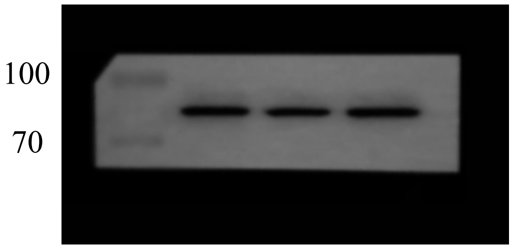

p-Akt (60 kDa)

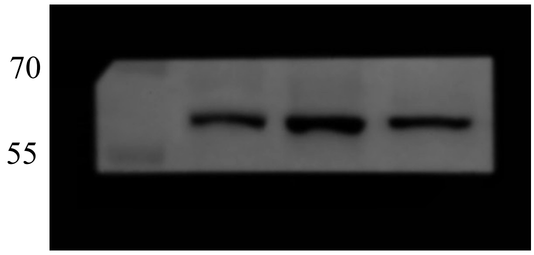

Akt (60 kDa)

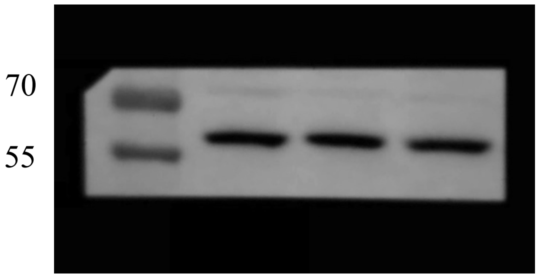

GLUT4 (53 kDa)

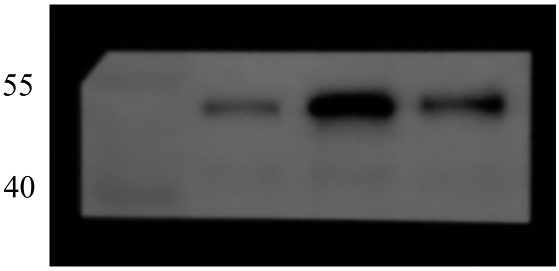

Actin (42 kDa)

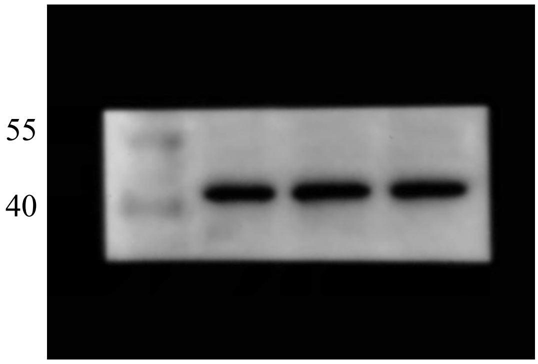

Fig. 5D repeat1

PINK1 (63 kDa)

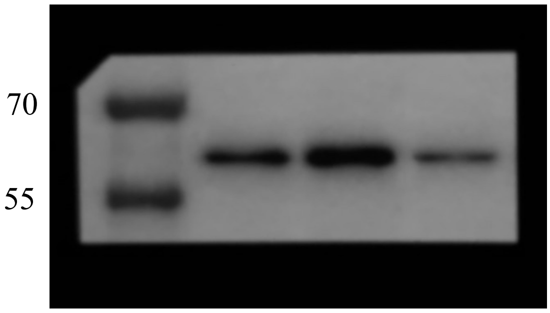

Parkin (50 kDa)

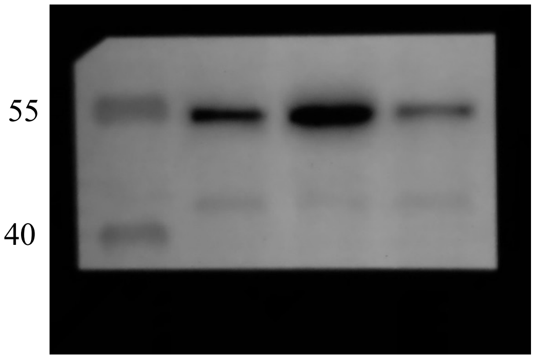

p62 (62 kDa)

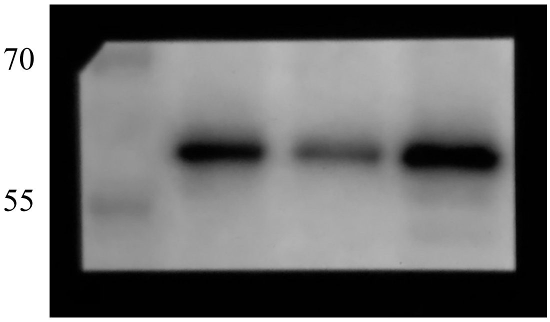

Actin (42 kDa)

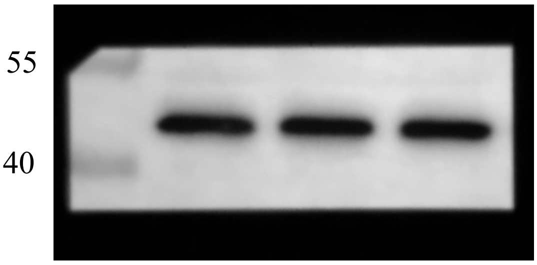

Fig. 5D repeat2

PINK1 (63 kDa)

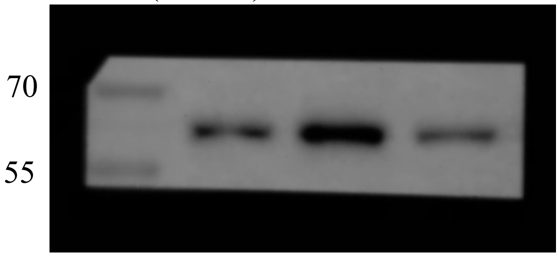

Parkin (50 kDa)

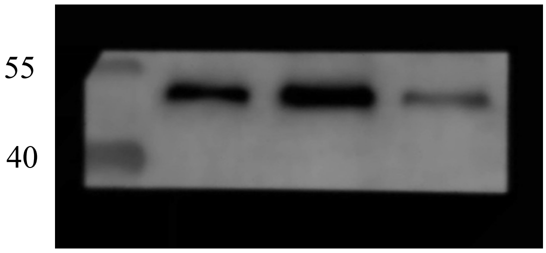

p62 (62 kDa)

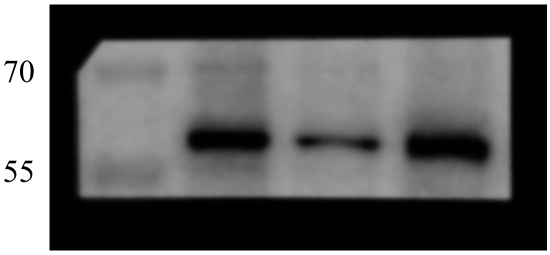

Actin (42 kDa)

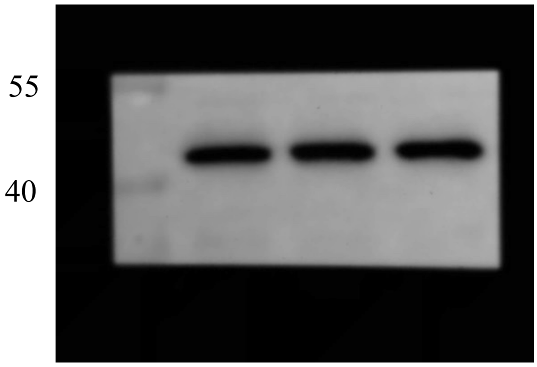

Fig. 5D repeat3

PINK1 (63 kDa)

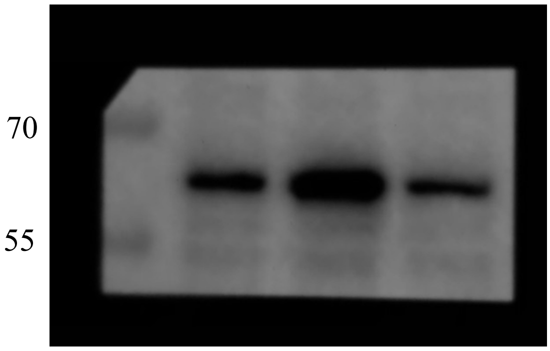

Parkin (50 kDa)

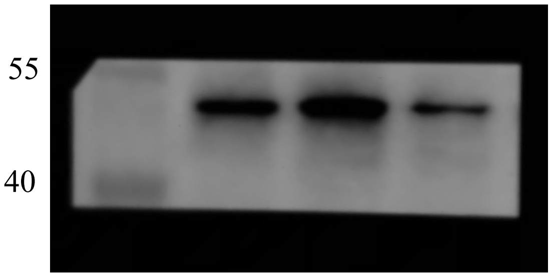

p62 (62 kDa)

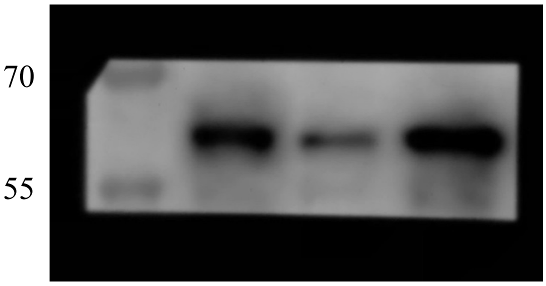

Actin (42 kDa)

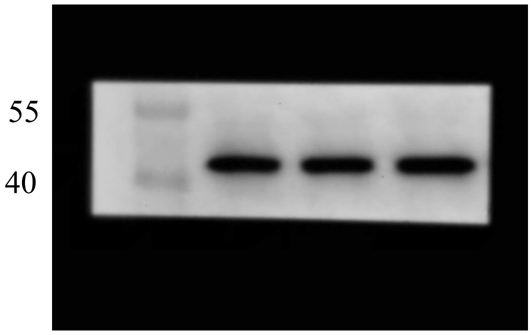

Supplement: Supplementary file 1 — Supplementary Material 1 [file 41598_2026_47924_MOESM1_ESM.pdf]
